# Transplant Trial Watch

**DOI:** 10.3389/ti.2023.11129

**Published:** 2023-02-01

**Authors:** Simon R. Knight

**Affiliations:** ^1^ Oxford Transplant Centre, Churchill Hospital, Oxford, United Kingdom; ^2^ Centre for Evidence in Transplantation, Nuffield Department of Surgical Sciences, University of Oxford, Oxford, United Kingdom

**Keywords:** kidney transplant, randomised controlled trial, immunosuppresion, diabetes mellitus, tacrolimus

To keep the transplantation community informed about recently published level 1 evidence in organ transplantation ESOT and the Centre for Evidence in Transplantation have developed the Transplant Trial Watch. The Transplant Trial Watch is a monthly overview of 10 new randomised controlled trials (RCTs) and systematic reviews. This page of Transplant International offers commentaries on methodological issues and clinical implications on two articles of particular interest from the CET Transplant Trial Watch monthly selection. For all high quality evidence in solid organ transplantation, visit the Transplant Library: www.transplantlibrary.com.

Randomised Controlled Trial 1 Criteria for Prediabetes and Posttransplant Diabetes Mellitus After Kidney Transplantation: A 2-Year Diagnostic Accuracy Study of Participants From a Randomized Controlled Trial.

*by Kurnikowski, A., et al. American Journal of Transplantation 2022 [record in progress]*.

## Aims

This post-hoc study aimed to investigate the diagnostic ability of fasting plasma glucose (FPG) and glycated hemoglobin (HbA1c) against the oral glucose tolerance test (OGTT)-derived 2-h plasma glucose (2hPG) in kidney transplant recipients (KTRs) from the Insulin Therapy for the Prevention of New Onset Diabetes After Transplantation study (ITP-NODAT).

## Interventions

Participants in the ITP-NODAT trial were randomised to either the basal insulin intervention group or the standard-of-care group.

## Participants

263 kidney transplant recipients (KTRs) from the ITP-NODAT trial.

## Outcomes

The main outcomes of interest were the evolution of posttransplant diabetes mellitus (PTDM), diagnostic accuracy of HbA1c and FPG criteria for PTDM and impaired glucose tolerance (IGT), and relationship of fasting plasma glucose and HbA1c versus 2hPG.

## Follow-Up

24 months after transplantation.

## CET Conclusion

This interesting post-hoc study investigates diagnostic parameters for post-transplant diabetes in a cohort of patients from the ITP-NODAT study. The authors demonstrate that around 1/3 of patients switch glycaemic category (normal/impaired glucose tolerance/diabetes) in the 2 years post-transplant. Use of conventional HbA1C or fasting glucose thresholds for diagnosis missed up to 69% cases diagnosed by a formal 2-h oral glucose tolerance test (OGTT). There are some limitations to this post-hoc study, including a relatively small sample size with few patients with PTDM, and a lack of data on ethnicity. However, it does demonstrate the usefulness of a formal OGTT in diagnosing post-transplant diabetes.

## Trial Registration

ClinicalTrials.gov—NCT03507829.

## Funding Source

Non-industry funded.

Randomised Controlled Trial 2 Prolonged-Release Once-Daily Formulation of Tacrolimus Versus Standard-of-Care Tacrolimus in *de novo* Kidney Transplant Patients Across Europe.

*by Budde, K., et al. Transplant International 2022; 35: 10225.*

## Aims

This study aimed to compare the posttransplant outcomes of LCP-tacrolimus (LCPT) versus current standard-of-care tacrolimus [immediate-release tacrolimus (IR-Tac) or prolonged-release tacrolimus (PR-Tac), according to centre preference] in *de novo* kidney transplant recipients.

## Interventions

Participants were randomly assigned to receive either LCPT or current standard-of-care tacrolimus.

## Participants

403 *de novo* kidney transplant recipients (≥18 years).

## Outcomes

The primary outcome was the tacrolimus total daily dose (TDD). The secondary clinical outcomes were treatment failure, treatment discontinuation, delayed graft function, local diagnosis of acute rejection requiring treatment, and concomitant immunosuppressive medications.

## Follow-Up

6 months.

## CET Conclusion

This phase IV multicentre study compared the use of LCP-tacrolimus with standard of care (either standard (SR) or prolonged release (PR) tacrolimus depending on centre preference) in *de novo* kidney transplant recipients. The authors demonstrated that despite a significantly lower total daily dose in the LCP-tacrolimus group, there was no difference in trough levels or short-term clinical outcomes between groups. The study is fairly well-designed, although the decision to allow the control arm to receive SR or PR tacrolimus at centre discretion is slightly odd as the study is left underpowered to show a difference in comparison to either in isolation. It is not really clear if there is any clinical benefit to an overall dose reduction; trough levels are similar so overall exposure is likely to be equivalent. Certainly, the study provides confirmation that the LCP-tacrolimus formulation is safe and equivalent in clinical efficacy to SR and PR formulations.

## Jadad Score

3.

## Data Analysis

Modified intention to treat.

## Allocation Concealment

Yes.

## Trial Registration

ClinicalTrials.gov—NCT02432833.

## Funding Source

Industry funded.

## Clinical Impact Summary

Tacrolimus has become the calcineurin inhibitor (CNI) of choice for maintenance immunosuppression following solid organ transplantation, demonstrating lower risk of acute rejection and improved graft survival compared to cyclosporine (1). It does have some drawbacks, including an increased risk of new-onset diabetes and an unfavourable pharmacokinetic profile with a rapid peak and narrow therapeutic window.

There have been a number of attempts to produce a tacrolimus formulation with a flatter pharmacokinetic profile and less pronounced peak, allowing once-daily dosing. Such a profile may have potential to reduce toxicity by reducing peak levels, and once-daily dosing may have an impact on compliance by reducing pill burden. The most-recent of these formulations is LCP-tacrolimus, which is reported to increase bioavailability and reduce first-pass metabolism compared to earlier formulations (2).

In a recent, phase 4 multicentre study, Budde et al. investigated the role of LCP-tacro in 401 *de novo* kidney transplant recipients across 10 European countries (3). Recipients were randomised to receive LCP-tacro or “standard care,” which could be immediate-release (IR) or prolonged-release (PR) tacrolimus alongside basiliximab, mycophenolate and corticosteroids. The authors demonstrated a significantly lower daily tacrolimus dose for the LCP-tacrolimus group to achieve slightly higher trough levels, confirming the improved bioavailability seen in earlier studies. However, there were no significant differences in clinical outcomes including rejection rates, graft survival, graft function or toxicity.

This large study was well-designed and reported, with central block-randomisation stratified by site and use of a modified intent-to-treat analysis. Whilst reflective of real-world variation in practice, the decision to allow either IR or PR tacrolimus as standard of care does limit the conclusions somewhat, as there is insufficient power to compare LCP-tacrolimus to either alternative formulation in isolation.

In reality, this study is unlikely to have much impact on clinical practice. A reduction in daily dose of tacrolimus alone is not sufficient to justify switching to what is presumably a more expensive formulation, although no health economic analysis is presented. Extended follow-up would be required to see if there is any benefit to the flattened pharmacokinetic profile on the risk of CNI toxicity in the longer-term.
